# Do Avoid Unnecessary Procedure in A Trauma Patient? A Case Report

**DOI:** 10.30476/BEAT.2021.89885.1238

**Published:** 2022-04

**Authors:** Mehdi Torabi

**Affiliations:** 1 *Department of Emergency Medicine, Kerman University of Medical Sciences, Kerman, Iran*

**Keywords:** Bronchi, Intubation, Multiple trauma, Pulmonary atelectasis

## Abstract

Endotracheal intubation is more commonly performed in the right main bronchus; however, it may rarely be performed in the left side. A 52-years-old man was brought to the emergency department by emergency medical services (EMS) after multiple trauma injury. There was a decrease in the right lung’s sound. Lung computed tomography (CT) scan revealed total pulmonary atelectasis. This scan was at the time that patient did not mention any recent history or complaint of pulmonary problems or diseases. In CT scan, we observed the white lung in the right side, the trachea which was deviated to the right, and the collapse-consolidation of the right lung was seen. The endotracheal tube image was observed in the left main bronchus which is a rare phenomenon. Decreasing of the right lung sound may not always be due to pneumothorax or hemothorax in trauma patients. In these patients, the rare phenomenon of left lung intubation should be considered as well. Left lung intubation may occur because of the lesion presence in the right lung.

## Introduction

Endotracheal intubation is a common procedure performed on patients with severe trauma referred to the emergency department. This procedure is more commonly performed in the right main bronchus; however, it may rarely be performed in the left side as well, which in this case, a decrease in the respiratory sound is heard on the right [1, 2]. In trauma patients, the sound decrease on the right side of the chest reflects hemothorax and pneumothorax, which requires faster performing emergency procedures when the patient’s condition is unstable such as needle decompression or tube thoracostomy of the chest cavity. In non-emergency situations, it is better to consider appropriate diagnostic interventions before performing any invasive procedure to prevent additional injuries to patients [3]. In this case report, we presented a patient with multiple traumas accompanied with respiratory sound decrease of the right lung due to the intubation of the patient’s left lung secondary to non-traumatic chest complications.

## Case Presentation

**Fig. 1:**
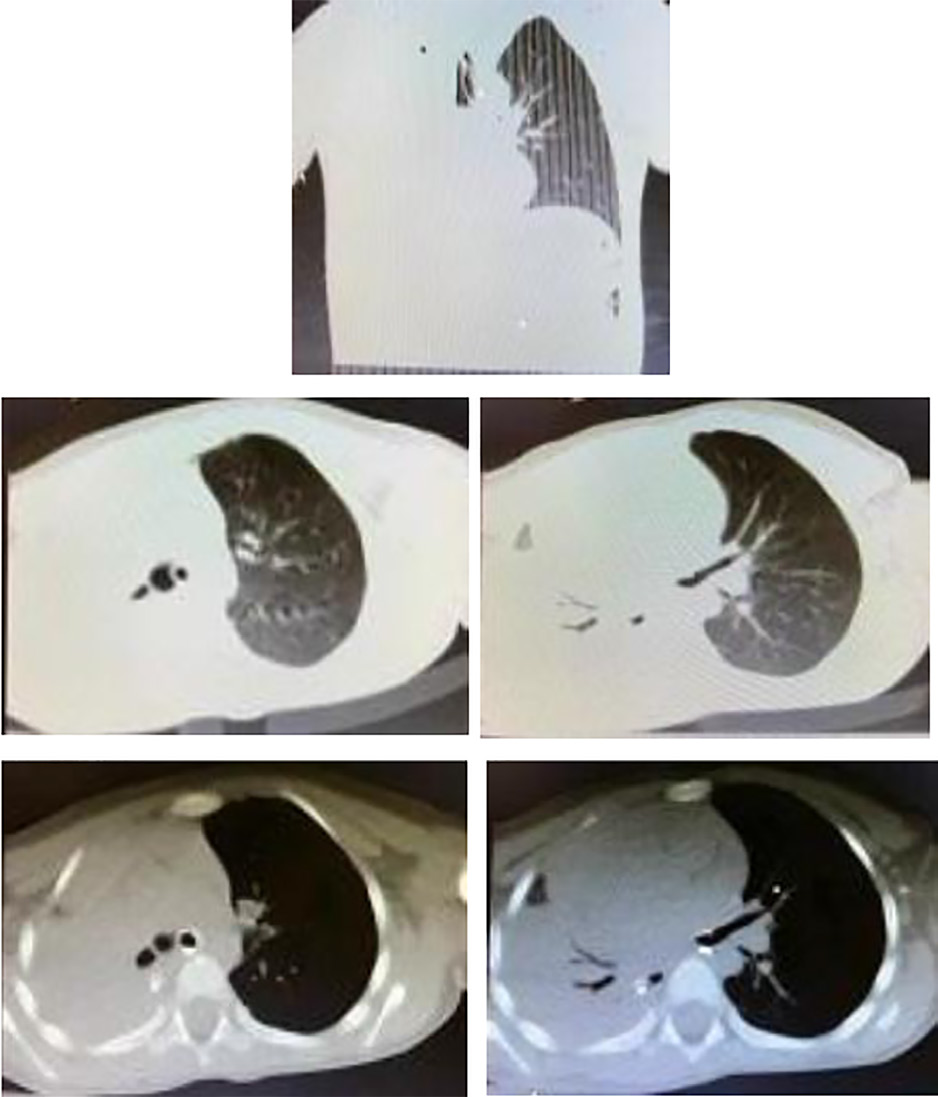
Lung Computed Tomography (CT) Scan of the patient

The patient was a 52-years-old man who was brought to the emergency department by emergency medical services (EMS) after a car accident. At the time of admission, he had confusion (GCS=8/15), and vital signs were including blood pressure (BP) 120/75 mmHg, heart rate (HR) 115, respiratory rate (RR) 18, and arterial oxygen saturation (O_2_ saturation) 88%. Orotracheal intubation was performed via the repetitive strain injury (RSI) method by using the tube No. 8 because of consciousness decreasing and the lack of airway protection. After intubation, the patients’ clinical examination showed a decrease in the right lung’s sound. There was no dilation in the neck veins. Focused assessment with sonography for trauma (FAST) ultrasound revealed no free fluids. Despite that the patient’s ventilation and oxygenation were appropriate, arterial O_2_ saturation was less than 90%. The diagnosis of pneumothorax or right hemothorax was considered because of decreasing in the right lung’s sound. As the patient’s vital signs were stable, a chest tube implant was eliminated. The patient underwent brain and lung CT scan while connecting to a portable ventilator. Lung CT scan revealed total pulmonary atelectasis, which this scan was at the time that patient did not mention any recent history or complaint of pulmonary problems or diseases. There was a view of the white lung in right side, the trachea was deviated to the right, and the collapse-consolidation of the right lung was seen. The image of the endotracheal tube was observed in the left main bronchus, which is a rare phenomenon (Figure 1). After 72 hours, the patient’s endotracheal tube was removed and the patient was referred to the surgical ward. During consultation with a pulmonologist, the patient was transferred to the lung ward for further investigation of right lung collapse.

## Discussion

Although endotracheal intubation in the right bronchus is seen in up to 12% of patients who undergo intubation, its incidence in the left side is rare [4, 5]. This is because that the right main bronchus is straighter (i.e., 45-degree angle to the vertical axis of the chip which is higher than the angle (25-degree) of the left bronchus to the vertical axis of the chip). Nevertheless, intubation may rarely occur in the left main bronchus. One reason for this phenomenon is the inappropriate position of the endotracheal tube and laryngoscope during intubation. The probability of left endobronchial intubation increases by inverting either the endotracheal tube (a 90-degree counterclockwise turn from the normal position and a change in the concavity of the tube to the left) or the laryngoscope [2, 6]. Nevertheless, the position of the tube and the laryngoscope during intubation was appropriate in the case presented in this study. Despite this, the diagnosis of pneumothorax or hemothorax was highly expected. Lung CT scan showed extensive pulmonary collapse-consolidation on the right side. Pulmonary collapse secondary to the obstruction of the right main bronchus triggered left bronchus intubation and a decrease in the right lung’s sound. The reason for the case becoming complicated was that he was referred as a trauma patient and that the patient’s companions mentioned no history of pulmonary lesions or problems. Therefore, in all patients, whether traumatic or not, all possible reasons for decreased right lung sound (such as collapse and its etiologies) should be considered. There are three types of collapse related to underlying diseases: the luminal which results from either the aspiration of a foreign object or mucus plaque, the mural which is caused by bronchogenic tumors, and finally the extrinsic which is resulted from the compressive effects of an adjacent mass [7]. In our patient, the decrease in the sound of the right lung was caused by the right-side collapse-consolidation and subsequent intubation into the left main bronchus. Therefore, decreased right lung sound in trauma patients may not always indicate hemothorax or pneumothorax, and other possible reasons for this event should be investigated in patients with stable hemodynamic condition. A well history-taking may partly help to reach an exact diagnosis; nevertheless, this was not true for our patient.

## Conclusion

Decreased right lung sound in trauma patients may not always be due to pneumothorax or hemothorax. In these patients, the rare phenomenon of left lung intubation should be considered as well. Left lung intubation may occur because of the presence of a lesion in the right lung. Therefore, it is possible to avoid unnecessary procedures by taking into consideration other possible causes of lung injury.

## Declaration

### Ethics approval and consent to participate:

The study was approved by the Ethics Committee of Kerman University of Medical Sciences.

### Consent for publication:

Oral consent was taken from patient guardian for participation in the study.

### Conflict of interests:

The authors declare no conflict of interest.

### Funding:

The present study was supported by Kerman University of Medical Sciences, Kerman, Iran.

### Authors' contributions:

Author contributed as follow to the conception or design of the work; the acquisition, interpretation of data for the work; and drafting the work or revising it critically for important intellectual content. The author approved the version to be published and agreed to be accountable for all aspects of the work.

### Acknowledgements:

We are grateful to all those who helped and supported us to write the manuscript.
